# A preliminary clinical report of transvaginal natural orifice transluminal endoscopic Sacrospinous Ligament Fixation in the treatment of moderate and severe pelvic organ prolapse

**DOI:** 10.3389/fsurg.2022.931691

**Published:** 2022-07-29

**Authors:** Zhenyue Qin, Zhiyong Dong, Huimin Tang, Shoufeng Zhang, Huihui Wang, Mingyue Bao, Weiwei Wei, Ruxia Shi, Jiming Chen, Bairong Xia

**Affiliations:** ^1^ Dalian Medical University, Dalian, China; ^2^ Department of Gynecology, The Affiliated Changzhou No. 2 People's Hospital of Nanjing Medical University, Changzhou, China; ^3^ Department of Gynecology, The First Affiliated Hospital of USTC, Division of Life Sciences and Medicine, University of Science and Technology of China, Hefei, China

**Keywords:** pelvic organ prolapse (POP), transvaginal single-port laparoscopy, sacrospinous ligament suspension, V-NOTES, surgical research

## Abstract

**Objective:**

To study the efficacy and safety of transvaginal natural orifice transluminal endoscopic Sacrospinous Ligament Fixation in the treatment of moderate and severe pelvic organ prolapse.

**Design:**

Patients were selected into this study on a voluntary basis to evaluate the short-term efficacy of this surgery by comparing the OP-Q scores before the operation, three months after the operation, and six months after the operation.

**Setting and Patients:**

Evaluate the clinical efficacy and safety by a retrospective analysis of the clinical data of the 18 patients with POP-Q grade III–IV pelvic organ prolapse treated by the Department of Gynecology of Nanjing Medical University Affiliated Changzhou No.2 People's Hospital from April 2020 to November 2020, and their post-operation follow-ups.

**Interventions:**

Patients with postoperative follow-ups found no obvious relapse without intervention measures.

**Measurements and Main Results:**

The transvaginal natural orifice transluminal endoscopic Sacrospinous Ligament Fixation was performed successfully, and the anterior and posterior walls of vagina and/or trans-vaginal hysterectomy were repaired as appropriate. Except the total vaginal length (TVL), the *P* values of numerical analysis for all points before, three months after, and six months after the operation were all <0.05, being statistically significant.

**Conclusion:**

This method is effective in the treatment of moderate and severe pelvic organ prolapse with few complications, but more cases and longer-term follow-up data are needed to determine the long-term effect of this procedure. For the selection of puncture sites, more anatomical data are needed to get more accurate result.

## Introduction

With the age increases, the incidence of pelvic organ prolapse increases significantly among women, especially for those who have delivered many times. Studies have shown that about 4.1% of pelvic prolapse patients aged 80 and above have clinical symptoms, which affect their quality of life (1–4). Research has indicated that about 11% of women below 80 underwent operation because of pelvic organ prolapse or stress urinary incontinence (5). While China is facing the challenge of an aging society, many other countries in the world, such as Japan and the United States, have already faced or are about to experience the arrival of an aging society (6, 7). At present, there are a variety of treatment methods for pelvic organ prolapse. For patients with clinical symptoms after failure of non-operative treatment, and who are unwilling to accept non-surgical intervention, surgical intervention has become the main option. The principle of treatment is to restore the normal anatomical structure of the pelvic floor. Since the sacrospinous ligament suspension was invented by Sederl in the 1950s, the operation has received constant improvements. Some studies have shown that the five-year failure rate of sacral ligament suspension is as high as 70.3% (8), but this is quite different from the about 37% failure rate reported by most studies (9–12). The author believes that the difference of postoperative recurrence rate may be caused by some factors, such as poor visual field of vaginal surgery, poor visual field of puncture during Sacrospinous Ligament Fixation, and different level of surgeons. Transvaginal natural orifice transluminal endoscopic (V-Notes) surgery has a history of years. The security and feasibility of these two methods have been proved. Therefore, the author believes that under the premise of good surgical skills and meticulous operation, the surgical method may be safe, feasible, and appropriate. V-Notes has gradually replaced some vaginal surgery because of its advantages of being minimally invasive, beautiful, and visualized. To this end, the author team has designed the transvaginal natural orifice transluminal endoscopic Sacrospinous Ligament Fixation. The good clinical results are reported as follows:

## Data and methods

### General data

18 patients with pelvic organ prolapse were selected from April 2020 to November 2020 in the Nanjing Medical University Affiliated Changzhou No. 2 People's Hospital. All patients were diagnosed according to the definition set forth in the ACOG guidelines, namely pelvic organ prolapse refers to the decline of one or more aspects of the vagina and uterus: the anterior wall of the vagina, the posterior wall of the vagina, the uterus (cervix), or the top of the vagina (vaginal vault or cuff scar after hysterectomy) (13). The average age of the patients was 62.61 ± 10.26 years old, and the average number of parturition was 2.00 ± 0.77. Each patient was scored and recorded by POP-Q after admission (14). Among them, 12 cases were Grade III and 6 cases were Grade IV. (Details are shown in Table 1.)

**Table 1:** Basic information of 18 patients.

Number	BMI (kg/m^2^)	Age (years)	Admission blood pressure (mmHg)	Reproductive history	Time for symptoms of prolapse
1	25.10	50	166/111	2-0-1-2	5 months
2	24.03	68	111/68	3-0-1-3	10 years
3	22.58	64	128/64	1-0-2-1	4 months
4	22.64	73	144/81	2-0-0-2	6 years
5	23.94	58	142/88	2-0-1-2	5 years
6	23.24	54	144/97	3-0-0-3	6 months
7	19.63	48	112/81	2-0-2-2	3 years
8	22.03	72	154/68	2-0-1-2	1 years
9	23.71	53	131/87	1-0-2-1	4 months
10	24.02	65	108/86	1-0-1-1	5 months
11	22.67	75	132/74	3-0-0-3	6 months
12	22.43	74	145/83	2-0-2-2	1 years
13	23.52	68	133/68	1-0-2-1	2 years
14	27.11	56	138/90	1-0-0-1	2 months
15	21.23	59	138/80	3-0-0-3	5 years
16	26.83	43	125/91	1-0-2-1	8 years
17	23.05	75	149/75	3-0-2-3	5 years
18	24.97	72	147/85	2-0-0-2	10 years

#### Case selection criteria

(1) Patients rated Grade III or above by POP-Q staging; (2) Patients who require surgical treatment after receiving non-operative treatment (Pessary, pelvic floor muscle training, etc.); (3) Patients who voluntarily accept this procedure and sign the informed consent form for the operation.; (4) Patients with good postoperative compliance and who can be followed up on time.

#### Case exclusion criteria

(1) Patients with severe medical complications who cannot tolerate surgery and anesthesia; (2) Patients with related surgical contraindications (such as acute reproductive tract infection, vaginal injury, and genital tract deformities such as vaginal stricture); (3) Patients with malignant tumors of pelvic and abdominal organs.

### Methods

#### Preoperative preparation

Explain the risks related to the procedure before operation, sign the informed consent form, prepare patients for the routine gynecological laparoscopy before operation, change them to fluid diet, and carry out vaginal disinfection three days before the procedure to reduce the probability of postoperative infection. Patients with atrophy of vaginal mucosa were treated with estrogen ointment to improve the vaginal environment before the procedure. Prepare vaginal surgical instruments and transvaginal single-hole laparoscopic instruments before operation.

#### Establishment of surgical approach platform

After the surgical area and vagina were disinfected, towels and indwelling catheterization were disinfected. The No. 1 silk thread was sutured on both sides to fix the bilateral labia minora to the root of the bilateral thighs to expose the surgical field. After the speculum is slowly inserted, the posterior lip of the cervix was clamped with cervical forceps and pulled upward to expose the posterior wall of the vagina. The wall was cut longitudinally in the middle and lower part, and the connective tissue in the vaginal rectal space was separated step by step, exposing the pelvic floor muscle and the adjacent tissue, so as to create the space for the operation and insert the special port for the single hole of the vagina.

#### Surgical instruments and materials

Stryker complete digital laparoscopic system, transvaginal single-hole protective cover and special port (Beijing Aerospace Cadi Company), one pair of conventional laparoscopic scissors, one needle holder, one ultrasonic knife, one attractor, one bipolar electrocoagulation forceps, two surgical separation forceps, one 30° conventional laparoscopic lens, one light source system and pneumoperitoneum system, one set of conventional surgical instruments, two Ethibond Excel W6937 non-absorbable sutures, and other absorbable sutures, silk thread, and so on.

#### Anesthesia and posture

Tracheal intubation general anesthesia was used in this operation. Before anesthesia, itinerant nurses assisted patients with the bladder lithotomy position (keeping head low, foot high ≥30°, legs abduction <90°). The posterior was about one punch beyond the operating table to provide space, and braces were placed on both shoulders to prevent slippage injury.

#### Surgical procedure

**Figure 1:**
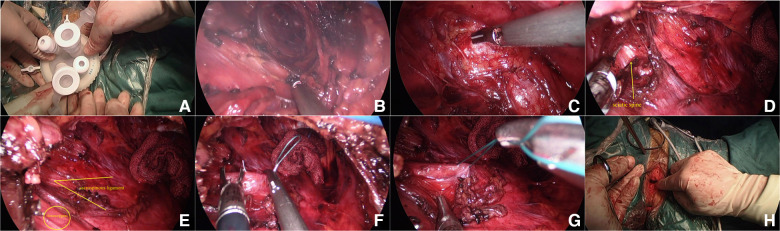
Surgical procedures. (**A**) Installing a specialized port for transvaginal single hole; (**B**) Separate tissue; (**C**) Expose the sacrospinous ligament at the sacrococcygeal attachment; (**D**) Expose the sciatic spine; (**E**) Expose the sacrospinous ligament and iliococcygeus; (**F**) Ethibond Excel W6937 non-absorbable suture to suture the sacral spine ligament; (**G**) Pull the suture to judge the tension; (**H**) Tie a knot with the suture.

For patients with anterior and posterior vaginal wall prolapse, anterior and posterior vaginal wall repair was feasible, followed by transvaginal single-hole laparoscopic Sacrospinous Ligament Fixation, and vaginal hysterectomy could be performed first for patients requiring hysterectomy. For patients with prolapse of the anterior vaginal wall, the anterior vaginal wall could be cut longitudinally to trim off part of the excess anterior wall tissue, and for patients with stress urinary incontinence, the bladder could be wrapped inside the anterior wall during purse suture after pruning to increase perineal pressure. Method of transvaginal single-hole laparoscopy: After exposing the surgical field, take the middle and lower part of the posterior wall of the vagina, longitudinally cut open the wall about 2.0–3.0 cm, separate a small airtight cavity with blunt fingers, and at the same time push the rectum to the left of the patient as far as possible to avoid intraoperative complications. Suture the skin flap around the incision, so as to prevent air leakage when tightening the purse after placing the protective sleeve of the incision. The incision protective sleeve is placed in the purse to open the incision and the closed space to make room for operation. Connect the incision protective sleeve to the specialized port of V-Notes to connect the pneumoperitoneum platform. Form the pneumoperitoneum by filling CO_2_ gas until the pressure reached 11 mmHg (1 mmHg = 0.133 kPa). Place a 30° laparoscopic lens into the operating hole to observe the visual field. Block the intestinal tube with a separation forceps to avoid injury, and then carefully separate the surrounding connective tissue with an ultrasonic knife to further enlarge the cavity. For beginners, the anatomical structure can be gradually separated to dissect the sacrum and the surrounding blood vessels and nerves, so as to avoid injuring blood vessels and nerves. After they get skilled, it is not necessary to expose too much tissue to locate the sacrospinous ligament. During the separation to enlarge the cavity, gauze can be used to stop the bleeding. The sacrospinous ligament can be located and the suture site of the sacrospinous ligament can be exposed through the landmark anatomical structures of the pelvic floor, such as sacrum, coccygeus, ischial spine, inferior gluteal blood vessel, and sciatic nerve. During the procedure, the ischial spine and sacrospinous ligament can be located by anal examination, and the suture site can be marked by bipolar after the position is determined. Two Ethibond Excel W6937 non-absorbable sutures can be used to suture the sacrospinous ligament with two stitches, with the depth being about 1/2 the thickness of the ligaments, and the distance between the two stitches being around 1 cm. After the needle is inserted, judge the tension by pulling the suture with the separation forceps without knotting. Then remove the port and tie the other end of the non-absorbable line with the suture at the top of the vaginal fornix of the hysterectomy patient, or tie it with the suture about 3 cm from the cervical orifice of the inferior wall of the cervix (that is, the uterine-sacral ligament close to the cervix) of the patient whose uterus was not removed after the repair of the anterior and posterior wall of the vagina. After tying the knot, lift the cervix with cervical forceps to detect whether it was fixed at the level of sacrospinous ligament. Suture the incision of the posterior wall of the vagina to end the operation. After vaginal disinfection, place a piece of iodophor gauze at the site and remove it the next day. (For detailed steps, see Figures 1A–1H.)

#### Postoperative management and follow-up

All the 18 patients returned to the ward safely after operation and underwent ECG monitoring within 24 h, with close attention paid to the postoperative vital signs and continuous low-flow oxygen inhalation. They received vagina disinfection on a daily basis during the three days after operation. After operation, the patients were immobilized in bed for 4 to 6 h. (The purpose was to prevent them from falling due to the residual effect of anesthesia. When patients were awake enough to get out of bed and walk around, we encouraged them to get out of bed as soon as possible to avoid the formation of venous thrombosis of the lower extremities.) They were given antibiotics to prevent infection and, if necessary, analgesics and sedatives. The outpatients were followed up in the 3rd, 6th, and 12th months, and their POP-Q scores were measured and recorded. Among the 18 patients, one who underwent operation on May 12, 2020, reported dull pain in both lower limbs two months after surgery, and another patient who underwent operation on November 9, 2020, reported lumbosacral distension sensation on November 30. Both of them have recovered. During the postoperative follow-up in the 6th month, one patient reported slight distension of the anterior vaginal wall. Only the data of the 3rd and 6th month follow-ups were analyzed because some of the patients underwent operation less than 12 months ago. (Details are shown in Table 2.)

**Table 2:** Information related to patients’ surgery.

Number	operation time (min)	Intraoperative bleeding volume (ml)	HGB before operation (g/l)	HGB after operation (g/l)	Postoperative hospital stay (days)	VAS pain score (Before operation)	VAS pain score (Three months after operation)
1	275	100	125	96	10	5	1
2	205	80	140	141	6	4	1
3	125	100	122	116	5	5	1
4	175	100	139	118	6	5	1
5	175	80	126	116	6	5	1
6	200	100	138	116	9	5	2
7	160	150	124	89	5	4	1
8	170	500	94	104	13	5	2
9	145	50	132	125	9	5	0
10	200	300	141	92	12	4	1
11	180	100	120	106	7	5	1
12	250	200	108	100	7	5	1
13	190	100	131	107	9	5	1
14	215	80	142	126	8	5	1
15	145	80	129	118	7	5	1
16	225	50	136	116	5	5	0
17	195	50	142	117	11	5	1
18	240	200	134	106	8	5	1

### Statistical analysis

The data of this study were statistically analyzed by SPSS21.0 statistical software. The measurement data were expressed by (mean ± standard deviation). The POP-Q scores of preoperative and postoperative follow-ups were tested by paired sample t-test, and the difference was considered to be statistically significant when *P* is <0.05.

## Results

All the 18 patients completed the operation successfully and underwent transvaginal single-hole laparoscopic Sacrospinous Ligament Fixation. As per the individual conditions and wishes, anterior vaginal wall repair, posterior vaginal wall repair, transvaginal hysterectomy, or V-Notes adnexectomy was carried out. A total of 2 patients underwent Mann's operation at the same time, and 14 received vaginal hysterectomy at the same time. The adjacent pelvic organs were not injured during the operation, and the patients were able to urinate on their own after the catheter was removed after operation. The operation duration was (192.78 ± 38.81) min, and the amount of blood loss was (134.44 ± 111.21) ml. Except for the total vaginal length (TVL), the *P* values of numerical analysis for all points before, three months after, and six months after the operation were all <0.05, being statistically significant. (Details are shown in Tables 3 and 4.)

**Table 3:** POP-Q scores before and 3 months after operation.

Group	Number	Aa	Ba	Ap	Bp	C	TVL
Before operation	18	1.00 ± 1.00	2.31 ± 1.19	0.86 ± 0.97	1.64 ± 1.08	5.19 ± 2.18	8.72 ± 0.46
3 month after operation	18	−2.25 ± 0.24	−2.68 ± 0.15	−2.34 ± 0.16	−2.68 ± 0.13	−7 ± 0.42	7.31 ± 0.57
*t*	** **	13.41	17.67	13.87	16.82	23.27	8.18
*p*	** **	0.014	0.001	0.005	0.001	0.001	0.559

**Table 4:** POP-Q scores before and 6 months after operation.

Group	Number	Aa	Ba	Ap	Bp	C	TVL
Before operation	18	1.00 ± 1.00	2.31 ± 1.19	0.86 ± 0.97	1.64 ± 1.08	5.19 ± 2.18	8.72 ± 0.46
6 month after operation	18	−2.17 ± 0.45	−2.52 ± 0.17	−2.32 ± 0.20	−2.55 ± 0.23	−7 ± 0.41	7.31 ± 0.57
*t*		12.25	17.03	13.67	16.07	23.27	8.18
*p*		0.037	0.001	0.007	0.001	0.001	0.559

## Discussion

POP has a variety of risk factors, such as age, hysterectomy, obesity (BMI > 30 kg/m^2^), smoking, long-term chronic Valsalva (cough, fatigue and weightlifting) stimulation, history of multiple births and vaginal delivery, and genetic defects in pelvic floor support (13, 15). The average age of 18 patients in this study was 62.61 ± 10.26 years, the average number of parturition was 2.00 ± 0.77, the average BMI was 23.49 ± 1.81 kg/m^2^, and no patient had BMI > 30 kg/m^2^. In POP, when the organ is still above the hymen, the most obvious symptom is the sensation of swelling and pressure in the vagina, followed by the influence of urination and defecation function. It has been reported that the incidence of POP with symptoms is between 3% and 12% (1). According to statistics, with the POP staging method, 40% of the patients were diagnosed Grade II or above, but only 10% - 20% of the patients went to the hospital (16). Since 1992, with DeLancey's three levels of vaginal support theory, namely the Level I apical vaginal support, Level II midvaginal support, and Level III distal vaginal support (17), gynaecologists have had more theoretical support for the treatment of POP. However, there is no uniform standard for the treatment, and nor is there any guide or expert consensus to clearly indicate which regimen is the gold standard for the treatment of POP. Pelvicfloormuscletraining (PFMT) is recommended for the prevention and treatment of POP (18), especially for patients with mild prolapse where PEMT can significantly improve the POP symptoms. Meanwhile, PEMT has been proposed to enhance the surgical effect of POP patients (19), but there is also literature concluding that PFMT cannot improve the results of surgical treatment (20–22). As a non-operative method for the treatment of symptomatic POP, Pessary is suitable for patients with surgical contraindications (23–26), but patients should find a suitable uterine support (27). Meanwhile, regular follow-ups should be conducted to monitor the contraindications of uterine support, such as vaginal atrophy, vaginal mucosal ulcer, erosion, and active vaginal vulvar infection. Mesh has also been one of the important methods for the treatment of pelvic organ prolapse, but now it is still a controversial topic whether to use mesh implantation to treat pelvic prolapse. Sacrocolpopexy (SC) is a surgical method for repairing horizontal defects at Level I. SC uses the anterior sacral ligament as the posterior anchor point to repair the vaginal axis. It is generally believed that suspending the vagina to the sacral promontory can avoid the formation of intestinal prolapse in the posterior pit. Abdominal sacrocolpopexy (ASC), Laparoscopic sacrocolpopexy (LSC) and Robotic-assisted Abdominal Sacrocolpopexy all have good therapeutic effects (28–36). Transvaginal single-hole laparoscopy can also be used in mesh-related surgery (37–39). But what cannot be ignored is the incidence of postoperative complications, especially the incidence of mesh erosion (40–42).

Repairing the three levels of support is the basic idea of more surgical treatment of POP. It has been reported that if only the anterior and posterior vaginal wall repair is completed, but the apical support is insufficient, there is still a possibility that POP repair fails (43, 44). Therefore, apical support repair is the most basic and important for the repair of pelvic organ prolapse. The use of patient's own tissue suspension can reduce the cost of surgery while reducing mesh complications (45–47). Here are four types of surgery to improve the apical support repair of patients' own tissue, which are widely used. (1) Modified McCall culdoplasty: Absorbable suture was used to suture one side of the hysterosacral ligament to the Douglas fossa and vaginal stump, and then suture the contralateral uterine sacral ligament. (2) Uterosacral ligament suspension (ULS): The rectovaginal fascia and pubic cervical fascia are suspended on the strong part of the uterosacral ligament. The part of the uterine-sacral ligament suspended from or above the ischial spine usually provides sufficient vaginal length and support. Shull suspension: Three stitches were sutured on each side of uterosacral ligament with 0 delayed absorbable suture, and the lowest suture was at the level of ischial spine. Three stitches were made upward at an interval of 1 cm, totaling fix sutures on both sides (48, 49). (3) Iliococcygeus fixation (ICF) is to fix the top of the vagina to the iliococcyx muscle and its fascia, usually suspended bilaterally. (4) Sacrospinous Ligament Fixation (SSLF): The sacrospinous ligament is used as the anchor point to suspend the vaginal fornix, and the right sacral ligament suspension is often performed, especially for patients with fornix prolapse. Through long-term follow-ups and comparison of the four surgical methods, some scholars believe that there is no significant difference in recurrence rate between Shull suspension and modified McCall culdoplasty (50, 51). There was no significant difference in success rate and recurrence rate between ICF and SSLF in a prospective non-randomized case-control study (52). An OPTIMAL randomized trial result showed that there was no significant difference in anatomical and functional results between USL and SSLF (21). According to a multicentre randomized trial, there was no significant statistical difference in SSLF versus vaginal hysterectomy with ULS within five years after operation among female patients with uterine prolapse Stage 2 or higher (53). Some similar studies suggested that the main determinant of recurrence rate was preoperative POP-Q stage, the recurrence rate increased with the increase of POP stage, and there was no significant difference in postoperative recurrence rate between USLS and SSLF (54).

There is currently no unified regulation on the surgical methods for the treatment of pelvic prolapse, and there are about 100 surgical methods for the treatment of pelvic prolapse and related diseases. At this stage, the surgical methods for pelvic prolapse are highly individualized (55). However, it should be noted that each technique has its shortcomings. A retrospective study with a nine-year follow-up period found that there was no significant difference between modified McCall culdoplasty and SSLF recurrence rate of 15% (11). However, there is a risk of ureteral obstruction or injury in the modified McCall culdoplasty operation, and it is recommended to check the ureter during the operation. A prospective non-randomized controlled trial compared the efficacy and safety of ICF and ASC in patients with vaginal fornix prolapse with similar objective and subjective success rates. In the ICF group, the operation time was shortened, but the average blood loss was higher (56). As early as 1997, some scholars thought that the possibility of recurrence after SSLF was as high as 29%, requiring secondary intervention (57). A Danish national cohort study of Ipsilateral uterosacral ligament suspension (IUSLS) and SSLF found that the probability of recurrence after SSLF was higher than that after IUSLS (58). It has been reported that the incidence of postoperative anterior vaginal wall prolapse is high (59). This OPTIMAL randomized trial also found 13.7% recurrence of anterior wall prolapse exceeding hymen in SSLF group (21). Some scholars have proposed that the patients with wide genital hiatus have a higher recurrence rate after SSLF (60). The author believes that the recurrence rate of genital hiatus, SSLF is closely related to the exposure of the operative field, the accuracy of the puncture position of the suspended suture, and the operative experience. Excluding the surgical experience, the success rate of SSLF can be improved by improving the exposure of the surgical field and the accuracy of the puncture site.

In addition to the minimally invasive laparoscopic technology, another advantage is that it can greatly improve the surgical field and use high-definition lens and light source, so that surgical technique has seen rapid advancement. With the introduction of the Natural Orifice Transluminal Endoscopic Surgery (NOTES), this technique has quickly fueled the clinical exploration of this kind of surgery, and has become a hot topic in the field of minimally invasive surgery (61, 62). Some scholars confirmed that there were no surgical complications in the perioperative period, the standard one-month follow-up period, or the subsequent follow-up period (up to 14 months), concluding that V-Notes would not increase the risk of surgical infection (63). Based on this, the author team combines V-NOTES and SSLF and their respective advantages. The use of tough and non-extensible Sacrospinous Ligament can avoid the prolongation of traction caused by daily life, reduce the recurrence rate, and have little effect on the vaginal axis (64).

No matter what kind of operation is chosen, it is closely related to clinical anatomy. The proposal of the accurate medical treatment concept also echoes with the minimally invasive clinical surgery. How to more accurately complete the suspension of the sacral spine ligament, determine the length of the suspension position from the ischial spine and other anatomical structures and the depth of the needle, and how to avoid damage to the surrounding nerve plexus, blood vessels, rectum and so on, all require anatomic concepts for support. Most of the disadvantages of traditional postoperative complications such as postoperative pain and recurrent prolapse of transvaginal sacral ligament are caused by the inaccurate puncture site during the operation, the injury of the peripheral nerve plexus, or a too shallow or too deep suspension puncture site. If peripheral blood vessels are injured during operation, it is estimated that up to 1.9% of patients need blood transfusion treatment (65). This is a material departure from the minimally invasive concept, so we can see the importance of anatomy to precision medicine. The pelvic surface of Sacrospinous Ligament fits the coccygeus, but the Sacrospinous Ligament is thinner and tougher. The main nerves around it are lumbosacral trunk nerve, Posterior femoral cutaneous nerve, pudendal nerve, and small branches, with blood vessels such as arteriae glutaea inferior and arteriae pudenda interna. How to avoid the damage to these tissues is one of the keys to a successful operation. The average length of Sacrospinous Ligament is about 5.1–5.2 cm, and the thickness is about 0.2 cm. Because the upper and lower edges of coccygeus are beyond the range of Sacrospinous Ligament, the two are closely linked. If Sacrospinous Ligament is forcibly stripped, it will cause serious damage to coccygeus. Maldonado et al. put forward the concept of coccygeus sarospinous ligament (CSSL) (66). Because Sacrospinous Ligament is closely connected with coccygeus and anadesma, Hayashi et al. think that Sacrospinous Ligament is formed by the growth and development of coccygeus (67), and some scholars think that the suspension tissue selected by SSLF is very likely to be coccygeus of CSSL (68). However, the author believes that if the suture is too shallow, only coccygeus and its anadesma will be suspended, because its texture and extensibility may lead to a suspension failure, so the needle depth, angle, needle distance, and puncture site need to be well selected. Most of the Pudendal canal composed of pudendal artery, Pussy vein, and pudendal nerve were located on the dorsal side of the Sacrospinous Ligament. Because the rectum is located on the left, the right Sacrospinous Ligament is generally selected for SSLF to avoid rectal injury. Chinese reports suggest that the anatomical distance between the right Pudendal canal and the ischial spine is mostly about 1.51 cm, and the anatomical distance between the ischial spine and the ischial spine is about 2.1–2.5 cm. The data outside China is higher, about 2.1–2.5 cm or even 2.75 cm (63, 69, 70). The difference in value may be related to the race of the population, measurement errors, and individual differences of cadavers. According to the current data, SSLF in China should be at least 1.51 cm away from sciatic spine to reduce the probability of damage to the pudendal tube. From the anatomical data, it can also be concluded that the probability of damage to Sacrospinous Ligament can be reduced by selecting the 1/2 depth of the shallow layer of the Sacrospinous Ligament 2.5 cm away from sciatic spine (71). S3, S4 and pudendal nerve are mostly on the upper edge of Sacrospinous Ligament (72). Therefore, choosing the place near the inferior edge as the puncture point can reduce the probability of injury to these nerves. The distance between nervi ischiadicus and Sacrospinous Ligament is about 2 cm, and the probability of nervi ischiadicus injury during operation is small. However, the sciatica of some patients reported in the literature may be caused by traction of Sacrospinous Ligament during suspension, most of which can be relieved by themselves. It has long been confirmed that musculi levator ani is controlled not only by internal anal sphincter nerves but also by pudendal nerve branches (73). Therefore, the clinical manifestation of puncture injury after internal anal sphincter nerves is not obvious. To sum up, the best puncture position on the right side of Sacrospinous Ligament selected by SSLF is at least 2.5 cm from the ischial spine, close to the inferior edge of 2.5 cm, and the depth is about 1 mm, where it can avoid damage to the surrounding blood vessels and nerves as much as possible while providing enough strength for suspension.

In this study, the data was derived from 18 patients, as shown in Tables 1, 2. The average operation duration was 192.78 ± 38.81 min, which was longer than the reported 92.3 ± 31.5 min of 453 patients with SSLF (74), longer than the 122.8 ± 36.1 min of 57 SSLF patients (75), and longer than the 86.04 ± 28.70 min of 50 patients with H-SSLF (76). This outcome may be related to the increased difficulty of transvaginal single-port laparoscopic surgery. The intraoperative blood loss of this study was 134.44 ± 111.21 ml, which was higher than the 92.3 ± 91.4 ml of 453 SSLF patients (74) and 86.80 ± 91.44 of 50 H-SSLF patients (76). This outcome may be related to factors such as increased surgical difficulty, small sample size, and initial surgery. The mean preoperative hemoglobin in this study was 129.06 ± 12.64 g/L, the mean postoperative hemoglobin was 111.61 ± 12.96 g/L, and the mean decrease in preoperative and postoperative hemoglobin was about 17 g/L, similar to the reported Preoperative Hb: 12.3 ± 1.06 (g/dl), Postoperative Hb:10.4 ± 1.07 g/dl decrease (75). The mean post-operative hospital stay in this study was 7.94 ± 2.41d. According to the data in Tables 3, 4, the treatment efficacy for the 18 patients lasted till three and six months after the operation, without obvious recurrence. One patient received the operation on May 12, 2020, and was reported dull pain in February. Another patient received the operation on November 9, 2020, and was reported lumbosacral swelling in 2020. Both patients have improved themselves, considering that the symptoms of the two patients may be caused by small nerve injury from suture suspension.

The advantages of this method are as follows: (1) Laparoscopic operation under direct vision can reduce unnecessary injury. If there is accidental injury during operation, the laparoscopic mode is easier to deal with the issue. (2) For some patients who are difficult to complete SSLF, especially those with deep position of sacral ligament making the site locating in the vaginal mode difficult to complete, this scheme can better expose and distinguish the pelvic tissues such as sacral spine ligament and ischial spine, and locate the suspension puncture site more accurately to complete the suspension of sacral spine ligament. (3) Through the laparoscopic mode, it is easier for doctors to recognize the sacral ligament and to teach and inherit SSLF. The disadvantage lies in that (1) Because of the single-hole laparoscopic model, the operation cost may be higher than that of SSLF. It is believed that with the development of single-hole laparoscopic model and the reduction of port and other consumables costs, the gap between the surgical costs will also be narrowed. (2) The operator needs to adapt to the operation mode of V-NOTES, after which SSLF may become easier to locate the sacrospinous ligament under the laparoscopic mode.

As mentioned above, this study preliminarily concluded that this operation has good short-term effect and few complications in the treatment of moderate and severe pelvic organ prolapse, but the sample size of this study is small, and the follow-up period is less than 5 years. Therefore, more cases and longer-term follow-up data are needed to determine the long-term effect of this procedure. For the selection of puncture sites, more anatomical data are needed to get more accurate results.

## Data Availability

The raw data supporting the conclusions of this article will be made available by the authors, without undue reservation.
